# Outcomes of Osteoid Osteoma Treated by Percutaneous CT-Guided Radiofrequency Ablation

**DOI:** 10.7759/cureus.42675

**Published:** 2023-07-30

**Authors:** Anurag Bhakhar, Ajay Sharma, Raghavendra Kaganur, J Pragadeeshwaran, Nirvin Paul, Rakesh Kumar Dhukia, Meenu Bagarhatta, Narendra Joshi, Amit Mor, Aman Sachdeva

**Affiliations:** 1 Department of Trauma Surgery and Critical Care, All India Institute of Medical Sciences, Rishikesh, Rishikesh, IND; 2 Department of Orthopaedics, All India Institute of Medical Sciences, Patna, Patna, IND; 3 Department of Orthopaedics, SMS (Sawai Man Singh) Medical College, Jaipur, IND; 4 Department of Radiodiagnosis, SMS (Sawai Man Singh) Medical College, Jaipur, IND; 5 Department of Orthopaedics, Post Graduate Institute of Medical Sciences, Pandit Bhagwat Dayal Sharma University of Health Sciences, Rohtak, IND; 6 Department of Preventive Medicine, Post Graduate Institute of Medical Sciences, Pandit Bhagwat Dayal Sharma University of Health Sciences, Rohtak, IND

**Keywords:** bone tumor, proximal femur, numerical pain scale, osteoid osteoma, radiofrequency ablation

## Abstract

Introduction

Osteoid osteomas are the most frequent true benign bone tumor in the adolescent age group and the third most prevalent benign bone tumor overall. This study was designed to assess the effectiveness of the procedure and correlate it with the analgesia offered because of the significant burden of this illness and new literature supporting the successful outcomes of image-guided percutaneous radiofrequency ablation (RFA) in osteoid osteoma.

Methodology

This hospital-based interventional trial was carried out in a tertiary care referral center. Forty-two patients with osteoid osteoma, ranging in age from 9 to 30, were included in the study. The patients received RFA guided by computed tomography (CT), and they were postoperatively monitored at one, two, and four weeks and three, six months, and 12 months. A numerical pain scale (NPS) was used to evaluate the patient's pain both before and after the procedure. The preoperative and postoperative results were contrasted.

Results

A total of 42 participants were enrolled in the study. Eight (19.05%) women and 34 (80.95%) men made up the group. Complete pain alleviation (NPS=0) was attained in 42.8% and 96.4% of the study group in the first and second weeks post-procedure. Almost all patients began protected weight-bearing at one week, according to their level of pain tolerance. Osteoid osteoma of the talus was a remnant lesion in one patient that required further treatment after two weeks. During the duration of the follow-up, no problems were recorded.

Conclusion

Percutaneous CT-guided RFA of osteoid osteoma is a safe, minimally invasive procedure and greatly reduces the duration of hospitalization. It has excellent functional outcomes and no known complications.

## Introduction

Osteoid osteoma as a disease entity was first described by Jaffe in 1935 [1]. The prevalence of osteoid osteoma is high in the adolescent age range and ranks as the third-most common true benign bone tumor overall [2-4]. The femur and tibia are the most commonly affected bones in more than 50% of cases [5]. The tumor is most commonly found in the cortical and rarely in the cancellous, subcortical, or medullary areas of bone [4,6].

Osteoid osteoma patients typically report pain at the site of the lesion, which is worst at night and is relieved by aspirin. At the site of the lesion, higher concentrations of the COX enzyme and prostaglandin are found [7]. Osteoid osteoma is a great mimicker and can mimic a variety of conditions like monoarthritis of the hip when it arises from the intra-capsular proximal femur [8] and when the lesion is present in vertebrae, the patient may present with scoliosis [9]. Rarely is a biopsy necessary for confirmation of the diagnosis; instead, a computed tomography (CT) scan that can assess nidus size is the preferred investigation. The lesion may have an extensive amount of peripheral sclerosis and a core tiny nidus (less than 15 mm) [5]. Extensive edema can be seen surrounding the lesion on magnetic resonance imaging (MRI). When viewed under a microscope, the histopathologic appearance may include fibrovascular tissue and immature bone trabeculae with osteoblasts in the periphery [10]. This histologic finding is similar to osteoblastoma, which has larger lesions (usually larger than 2-2.5 cm) than osteoid osteoma [5].

There are numerous treatment options, including surgical removal of the tumor, percutaneous CT-guided radiofrequency ablation (RFA), and medical management with non-steroidal anti-inflammatory drugs (NSAIDs). The lesion may heal in three to four years if the patient's symptoms are well-controlled with long-term medical management, but the course is unpredictable and the outcome of conservative treatments varies in the literature [9]. An exact location of the nidus can be difficult to determine during surgery, and a substantial amount of bone will have to be removed, making quick mobilization and weight-bearing impossible in the majority of instances. Where RFA cannot be utilized, surgical excision is helpful, like in spinal osteoid osteoma [11]. Percutaneous image-guided RFA is a minimally invasive procedure with good safety, efficacy, and minimal hospital stay [12]. A more recent, radiation-free method called magnetic resonance-guided focused ultrasound (MRgFUS) is being tested for the treatment of osteoid osteoma [13].

Following these various treatment modalities (including surgical) for osteoid osteoma, pain is measured using a variety of tools, such as the visual analog score (VAS), the numerical pain score (NPS), etc. In the study, NPS (range 0-10) and VAS were used for pain measurement, both of which have a high correlation with other pain measurement tools [14,15].

## Materials and methods

A prospective interventional study was conducted at a tertiary care referral hospital in North India from February 2021 to February 2022. All newly diagnosed cases of osteoid osteoma with no prior surgical or radiological treatment were recruited in the study. Cases with recurrence and partially treated cases were excluded from the study. Ethical clearance for conducting the study was obtained from the Institutional review board and the approval number was 450/MC/EC/2021.

NPS and VAS were used for comparison of the severity of pain preoperatively and postoperatively for the effectiveness of treatment. Due to the nocturnal rise in pain that is typical of osteoid osteoma, separate NPS and VAS measurements were collected during the day and at night.

All patients were informed about various other modalities of treatment and detailed informed consent was taken in the local language from the patient or the parent (if the patient was below 18 years of age). Patients were followed up at one, two, and four weeks, and three, six, and 12 months, and VAS and NPS were documented on each follow-up. Before leaving the hospital, each patient underwent a physical examination to check for any issues linked to the treatment such as incision site bleeding, swelling, skin burns, neurovascular complications, and other issues.

Intervention technique

Pre-procedure

**Figure 1:**
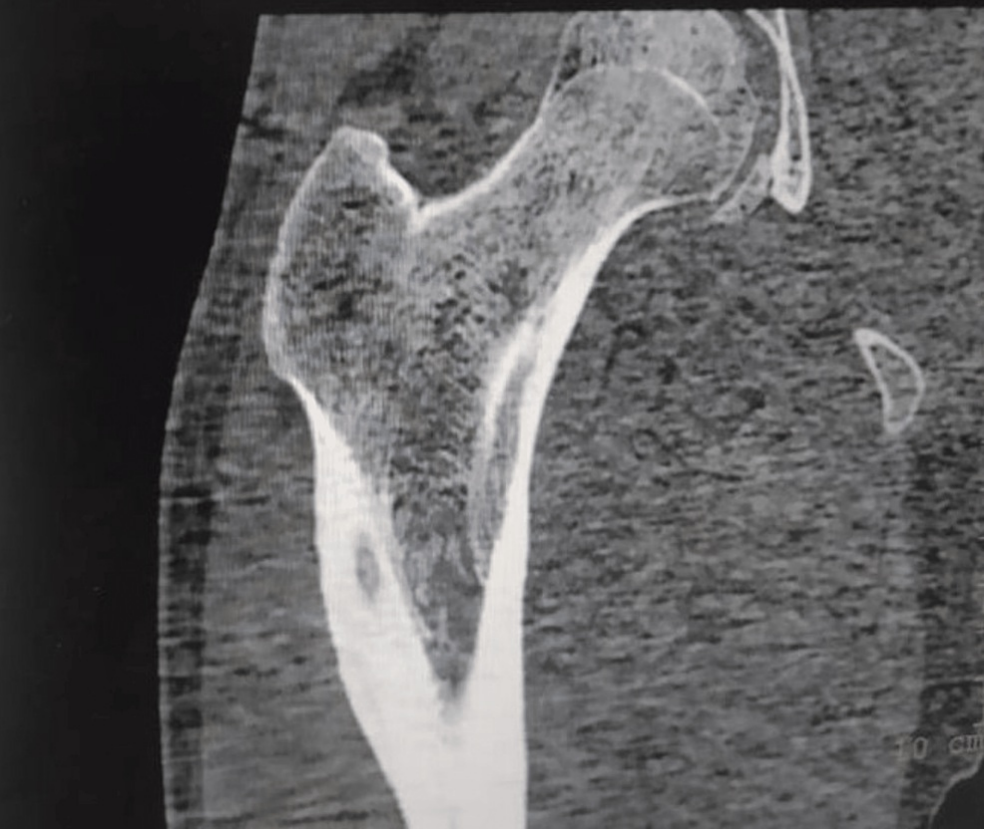
Coronal section of multidetector computed tomography scan of the proximal femur showing osteoid osteoma

Informed consent was obtained before the procedure. Dispersive grounding pads were applied in proper alignment, with good skin contact, and as close to the ablation site as possible, to allow the shortest current path through the patient. Regional anesthesia was administered in the form of spinal anesthesia for lesions in the lower limb and supraclavicular block was administered for lesions of the upper limb. Using a multidetector computed tomography (MDCT) scanner, the lesions were localized in thin sections (Figure 1).

Procedure

**Figure 2:**
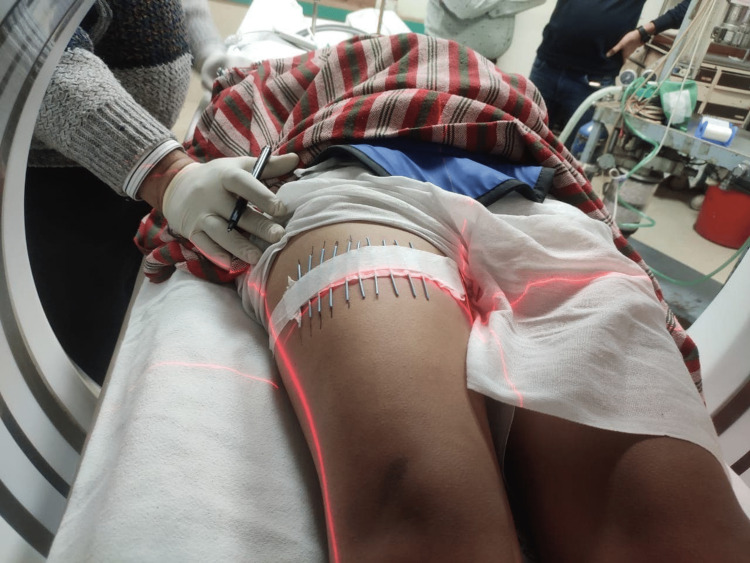
Radiopaque material placed over the lesion for the exact localization of the nidus for trochar placement

In our research, a Covidien Cool Tip E series radiofrequency generator (Dublin, Ireland) was utilized. Radio-opaque markers are placed on the skin overlying the lesion (Figure 2). For improved anchorage, the angle should be perpendicular to the cortical surface and pass through the unaffected opposite cortex.

The relationship with adjacent neurovascular structures was assessed. A trochar and cannula were introduced into the lesion under CT guidance. The trochar was retracted and under aseptic precautions, a 14-gauge RF electrode was introduced into the osteoid osteoma nidus. The cannula should be withdrawn more than 1 cm from the electrode tip before ablation.

**Figure 3:**
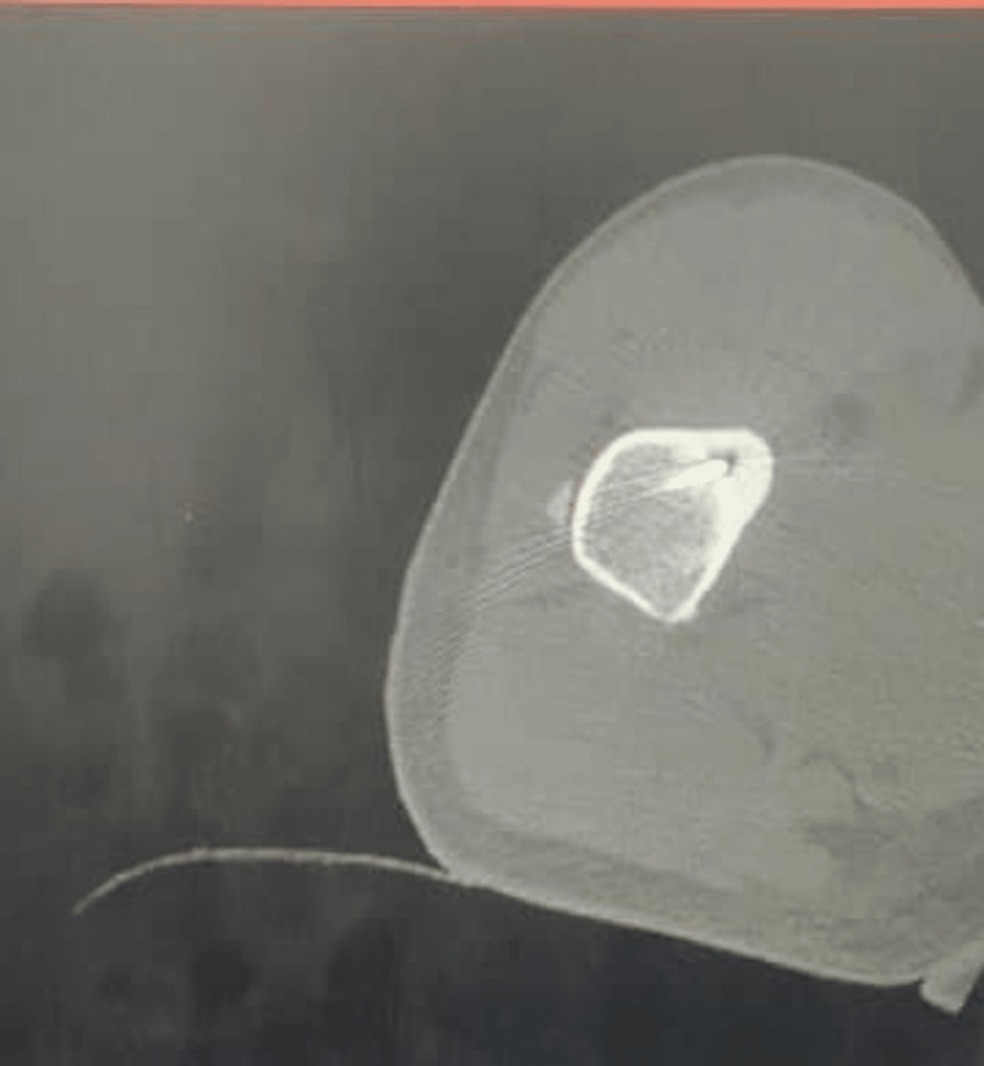
Axial section of multidetector computed tomography showing the radio frequency ablation probe inside the nidus

Spot pictures were captured to verify that the electrode and cannula were in an acceptable position (Figure 3). The tip temperature was raised to 90°C for six minutes after the grounding pad and electrode were attached to the RF generator. After the procedure, a small pressure dressing was applied at the percutaneous puncture site. All patients were discharged on postoperative day 2 after local site inspection.

## Results

A total of 44 patients with osteoid osteoma were received at our center. One patient had an incompletely treated lesion and one had a recurrence of the lesion after one year. Hence, both these patients were excluded from the study and a total of 42 patients were recruited. Nineteen patients (45.24%) were in the 9-15 year range, 15 patients (35.71%) were in the 16-20 year range, five patients (11.9%) were in the 21-25 year range, and three patients (7.14%) were in the 26-30 year range. The subjects ranged in age from 9 to 30, with a mean age of 16.17.

Thirty-four individuals in our study were male (80.95%) while eight (19.05%) were female. The cortical region, which was affected in 38 individuals (90.48%), was the most frequently affected place in the bone, followed by intramedullary and subperiosteal involvement in two (4.76%) patients each. The proximal femur was the site of the lesion in the majority of individuals (24 patients; 57.14%), followed by the shaft of the tibia with seven (16.67%) patients. The mean nidus size was 8.12±1.52 mm.

At one week and two weeks after the treatment, we saw comprehensive pain alleviation (NPS = 0) in 42.8% and 96.4% of the study population, respectively. Preoperative mean NPS for day and night were 7.67 and 8.35, respectively, indicating that nocturnal pain is more intense. The mean NPS for day and night was 0.92 and 0.55 (p-value = 0.001), respectively, on postoperative day 7 follow-up, indicating a considerable reduction in the intensity of pain and the efficacy of our treatment. Almost all patients began protective weight-bearing at one week, depending on their pain threshold.

On successive follow-ups, there was total pain alleviation, and up until the last follow-up (mean follow-up, 9.5 months), no patient had experienced any complications following RFA. Patients have been advised to follow up every six months for the first two years and once a year after that to look for recurrence.

## Discussion

Osteoid osteoma can be treated in several ways, including image-guided RFA, surgical excision, and MRgFUS [16]. In patients who experience total symptom alleviation, conservative therapy with NSAIDs can be taken into consideration. The course of the disease is unpredictable, and the prognosis may depend on the location of the tumor [17]. For example, the site of the tumor near the growth plate may cause angular deformity and limb length discrepancy, the intraarticular tumor may cause synovitis of the joint, and scoliosis can result from osteoid osteoma of the spine [18]. The affected bone portion is removed during surgical excision of osteoid osteoma; however, the accurate location of the tumor is challenging. The patient must adhere to postoperative immobilization and non-weight-bearing guidelines, and this technique occasionally necessitates intraoperative bone grafting. Therefore, given the current situation, surgical resection cannot be recommended as an effective mode of treatment for osteoid osteoma at the majority of sites. The preferred course of treatment for osteoid osteoma that is close to a vital structure, such as the spinal cord, is surgical excision [12]. In the current scenario, percutaneous CT-guided RFA is the treatment of choice because this procedure overcomes all the demerits of surgical resection of this tumor.

We noticed that the subjects ranged in age from nine to 30, with a mean age of 16.17, which was in line with previous studies in which almost 70% of patients with osteoid osteoma were under 20 years old [19]. In our research, men were more likely to have the condition than women, which was consistent with the literature's description of a 2:1 male-to-female ratio [4].

Osteoid osteoma commonly affects the cortices of the long bone's diaphysis, usually the lower limb, which was consistent with our findings [20]. Other locations noticed in our study were the shaft of the fibula, the proximal humerus, the proximal tibia, the distal femur, the distal tibia, the ischium bone, and the proximal phalanx of the ring finger also affected, with the last four locations having the fewest cases. In the study they conducted, Papathanassiou et al. found that more than half of osteoid osteomas impact long bones (the femur and tibia), with the proximal femur being the most commonly afflicted area [20]. Kransdorf et al. also noted the same finding concerning anatomical distribution [4].

Luigi De Palma et al. treated 20 patients with osteoid osteoma using the same modality and found that more than 95% of the time (p-value = 0.01), the patients experienced significant pain relief [21]. Bhavin Jankharia et al. performed RFA on 40 patients; 38 of them experienced pain reduction, and two of them experienced a recurrence [22]; however, when the same treatment was performed again on them, they experienced complete pain relief, so their study's primary and secondary success rates were 95% and 100%, respectively. In research by Santiago et al., the identical technique resulted in significant pain reduction for 14 of 15 patients, or 93% of them [23]. However, one patient with tibial osteoid osteoma experienced a recurrence, necessitating open surgery to remove the nidus.

In a study on percutaneous CT-guided drilling by Agashe et al., 23 patients - 13 men and 10 women, with a mean age of 10.03 years participated [24]. The researchers concluded that CT-guided drilling is a very effective alternative for those who cannot afford the RFA operation. After this surgery, they discovered that there was no recurrence (mean follow-up was 19.72 months). We observed that the mean nidus size was 8.12±1.52 mm. Similar results were seen in a study done by Karagoz et al., where the mean nidus size was 8.06 mm [25].

A more recent method that uses radiation-free MRgFUS is being tested. The results of this study closely resemble those of ours. MRgFUS has the advantage of being a radiation-free procedure, but this procedure has the drawback of resolving osteoid osteoma in MRI less effectively than MDCT. Francesco Arrigoni et al. conducted a study on MRgFUS, using this technology to perform 32 procedures of ablation of osteoid osteoma. Of the 32 patients treated, all pain was completely relieved [13].

There are several reported problems of image-guided RFA in the literature, including skin burn, infection, vasomotor symptoms, and hematoma [26,27], but none of these occurred in our study, except for local swelling at the operation site. As of the most recent follow-up, no further complications were reported. Therefore, our recommendation for the preferred method of treatment for osteoid osteoma is CT-guided percutaneous RFA. In our study, regional anesthesia was administered (spinal anesthesia for a lower limb procedure and supraclavicular block for an upper limb procedure), and the pain was adequately managed during the procedure and early postoperative period. Thereafter, the pain was managed with parenteral and oral analgesics. Some prior studies also suggest only general anesthesia for better intraoperative positioning and postoperative pain management for this procedure [27].

The small sample size (n=42) of our study was one of its drawbacks, as a larger sample size would have indicated better outcomes from this technique. Additionally, the short follow-up period (average follow-up period of 9.5 months) was not ideal because recurrence could happen even after a year. Cases of osteoid osteoma were not histopathologically validated in our investigation, as it was not necessary for diagnosis. Finally, we haven't performed RFA on osteoid osteomas in less common locations like the spine, ribs, or foot. The success rate at those sites was therefore not evaluated.

## Conclusions

Even though numerous procedures have been described for the treatment of osteoid osteoma, percutaneous CT-guided RFA of osteoid osteoma is very effective, has great functional outcomes, is safe and the least invasive, requires a short hospital stay, and has no known complications. Recent techniques are beginning to take off, such as radiation-free MRgFUS. The results are comparable, but long-term randomized control trials are required to prove one method is superior to the other.
